# Simultaneous ultraviolet and first near-infrared window absorption of luminescent carbon dots/PVA composite film†

**DOI:** 10.1039/c8ra09742a

**Published:** 2019-03-06

**Authors:** Melda Taspika, Fitri Aulia Permatasari, Bebeh Wahid Nuryadin, Tirta Rona Mayangsari, Akfiny Hasdi Aimon, Ferry Iskandar

**Affiliations:** Department of Physics, Faculty of Mathematics and Natural Sciences, Institut Teknologi Bandung Jalan Ganesha 10 Bandung 40132 Indonesia ferry@fi.itb.ac.id; Department of Physics, UIN Sunan Gunung Djati Bandung Jl. A. H. Nasution 105 Bandung 40614 Indonesia; Research Center for Nanosciences and Nanotechnology (RCNN), Institut Teknologi Bandung Jalan Ganesha 10 Bandung 40132 Indonesia; Department of Chemistry, Universitas Pertamina Jl. Teuku Nyak Arief, Simprug Jakarta 12220 Indonesia

## Abstract

Liquid Carbon Dots (CDs) were successfully synthesized by hydrothermal method using urea and citric acid as raw materials. TEM images confirmed that the CDs have a spherical shape with a homogeneous distribution. The as-prepared liquid CDs could absorb ultraviolet (UV) and first near infra-red (NIR) window simultaneously. However, the photoluminescence (PL) of the liquid CDs was damaged by their quenching effect. To overcome this issue, the liquid CDs were dispersed in poly(vinyl) alcohol (PVA) to fabricate the composite film. Herein, the dual-peak absorption properties of the CDs/PVA composite films were investigated for the first time. The composite films could maintain the simultaneous UV and first NIR window absorption property even after being preheated up to 200 °C, implying that the structure of CDs was well retained during the transition from the liquid to films. Daylight treatment for seven days produced minimum changes in the UV-vis and PL spectra, which indicates that the CDs/PVA film has more stable optical properties than the liquid CDs.

## Abstract 2

The simultaneous UV and first NIR absorption of the CDs/PVA composite could be maintained up to 200 °C, with minimum PL changes.

## Graphical abstract

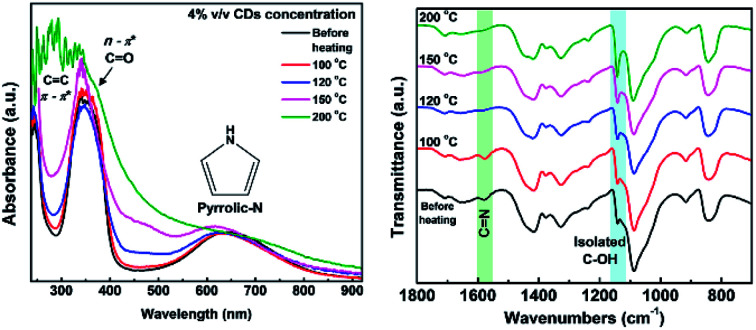

## Introduction

Carbon dots (CDs) are interesting phosphor materials that have attracted researchers' attention because of their outstanding properties such as high photostability, excellent biocompatibility, tunable photoluminescence, inertness, and the ability to combine easily with biomolecules.^1^ Currently, CDs have been applied in bioimaging,^2^ biosensors,^3^ photocatalysts,^4^ photothermal therapy,^5^ shape memory polymers,^6^ and optoelectronic devices.^7^ One of the conventional methods for synthesizing CDs is the hydrothermal method due to its low price, eco-friendliness, and ease of process.^8^ Besides, the hydrothermal method also enables the control of particle size, composition, purity, and energy consumption.^2^ However, the as-produced CDs by this method exist in the liquid form, which is prone to immediate damage by the quenching effect because of their interactions with the surrounding environment such as sunlight, water, O_2_, and H_2_O molecules. H_2_O molecules cause an increase in motion and rotational freedom of the liquid CDs, which induces self-assembly of the CD molecules.^5^ Therefore, in liquid CDs, aggregation or direct collision occurs easily between the CD molecules. The collision causes the CD molecules to lose their energy and become quenched.^9^ Surface functional groups, such as carboxylic and epoxide, are oxygen binding and cause non-radiative recombination of electron and hole, thus contributing to the quenching effect of liquid CDs.^10^ Carbonyl and epoxy groups generate non-radiative recombination of the localized electrons and holes in CDs.^11^ Non-radiative recombination is one of the causes of the quenching effect in CDs.^12^ Electrostatic force or electrostatic interaction between the CD molecules disturbs their electrostatic stability during agglomeration and thus causes quenching in liquid CDs.^5,13^

To resolve the quenching effect of CDs, liquid CDs were dispersed in a single matrix including silica,^14^ mesoporous alumina,^15^ and several polymers such as polyvinylpyrrolidone (PVP), polyacrylic acid (PAA), polyacrylamide (PAM),^16^ polymethyl methacrylate (PMMA), poly(*N*-isopropylacrylamide) (PNIPAM),^17^ and polyvinyl alcohol (PVA).^8,9,17–21^ All of these CDs/polymer composite films absorb only in the UV region. For medical applications such as photothermal and cancer therapy, the CDs/PVA composite films have to absorb in the first NIR window (650–900 nm) because the light in this region does not damage the living tissues.^22^ Previously, our group successfully synthesized liquid CDs that could absorb UV and first NIR window simultaneously and act as a photothermal agent.^23^ However, the as-produced liquid CDs were quenched several days after the synthesis. The quenching could have resulted from the damage of the pyridone structure induced by the interaction of CDs with sunlight.

PVA is a synthetic polymer that has great properties such as hydrophilicity, non-toxicity, excellent biological compatibility, great optical transparency, and simplicity in handling.^24^ These properties confer PVA with the potential for applications in light emitting diodes (LED),^18^ shape memory polymers,^6^ UV blocks,^21^ and photovoltaic devices.^9^ The hydroxyl groups of PVA aid hydrogen bonding with the surface functional groups of CDs. Besides, PVA also acts as a surface passivation agent that prevents the rotation and direct collision of the CD particles.^8^ For this reason, in this research, liquid CDs were dispersed in the PVA polymer, which acts as a matrix and a surface passivation agent. Hydroxyl groups of PVA interact with the surface functional groups of CDs through hydrogen bonding, which prevents the interaction of CDs with their environment. To the best of our knowledge, there is no report on CDs/PVA composite films that absorb UV and first NIR window simultaneously.

## Experimental

### Materials

All materials were purchased from Merck. Anhydrous citric acid (C_6_H_8_O_7_, Merck) was used as the carbon source and urea (CO(NH_2_)_2_, Merck) as the nitrogen source. Deionized water was used as the water source in the experiments. Polyvinyl alcohol (PVA) ([CH_2_CH(OH)]_*n*_, MW = 60 000, fully hydrolyzed >98%) was the matrix polymer in the composite films. All reagents were of analytical grade and not purified further before being used.

### Synthesis of liquid CDs

Liquid CDs were prepared according to our previous report,^23,25^ using urea (9 g) and anhydrous citric acid (6 g) as the raw materials. In brief, these starting materials were added to 100 mL of deionized water and stirred to form a transparent solution. Then the mixed solution was poured into an autoclave and heated at 160 °C for 5 hours. The obtained liquid sample was purified using a regenerated cellulose filter membrane with a pore size of 0.22 μm (Sartorius Stedim Biotech Co.) for further characterization. Then the sample was dispersed in the PVA solution without any pre-treatment.

### Synthesis of CDs/PVA composite films

The PVA solution was prepared by dissolving PVA granules (1 g) in 10 mL deionized water at 120 °C for 30 minutes and mixing continuously at room temperature for 30 minutes to produce a homogenized clear gel solution. Different volumes of liquid CDs (0.5; 0.8; 1; 2; 3; 4; and 5% v/v) were mixed with the PVA solution while stirring continuously at room temperature for 2 hours. The obtained homogeneous gel compound was poured on a glass substrate. The as-prepared precursor was heated in an oven at 60 °C for 2 hours. Finally, the CDs/PVA composite film was acquired and peeled off the substrate to produce a freestanding film. The film thickness was controlled during the fabrication by using the same amount of gel precursor and keeping the film dimensions as constant as possible.

### Characterization

The morphology and particle size of liquid CDs were investigated by transmission electron microscopy (TEM, Hitachi TEM running at 100 kV). The surface functional groups were confirmed by Fourier transform infrared spectroscopy (FTIR, Bruker ALPHA). UV-vis absorption and photoluminescence properties were studied using a self-assembled UV-vis spectrometer and a photoluminescence spectrometer from Agilent Technologies, respectively.

## Results and discussion

**Fig. 1:**
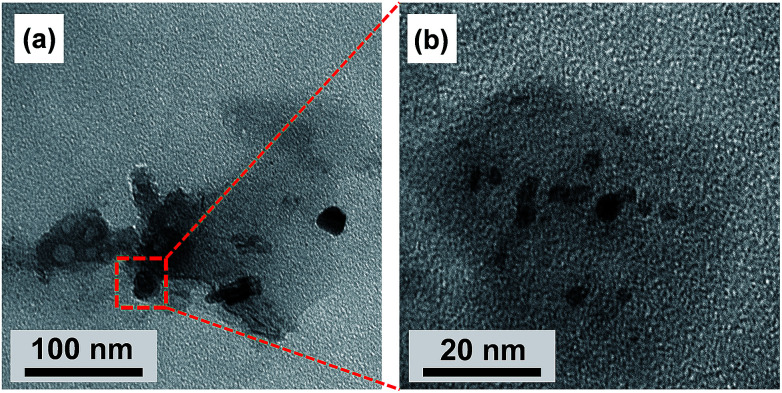
TEM images of liquid CDs: (a) low magnification and (b) high magnification.

Liquid CDs were synthesized using citric acid and urea as the raw materials. Fig. 1 shows the TEM images of liquid CDs. The low-magnification TEM image (Fig. 1a) shows a sheet formation in the as-prepared sample. CD formation started with the reaction of citric acid and urea, which formed citric acid amide. The nanosheet structure resulted from the self-assembly of citric acid amide due to dehydration and the deamination process of intermolecular compounds containing hydroxyl, carboxyl, and amino groups, which is induced by further heating under the hydrothermal condition.^25^ Several areas of the sheet consisted of spherical dots as confirmed by the high-magnification TEM image (Fig. 1b). The dots were well dispersed without agglomeration.

**Fig. 2:**
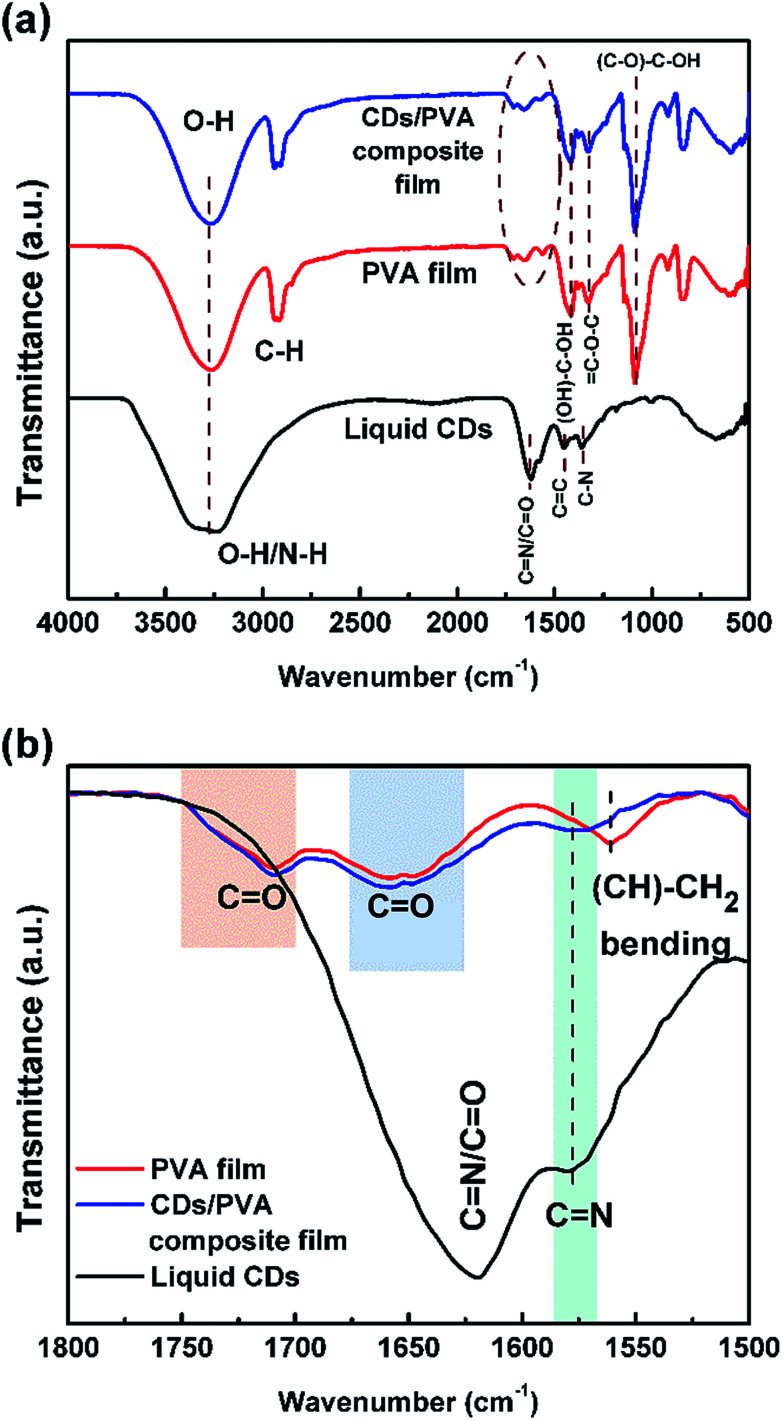
FTIR spectra of (a) liquid CDs, PVA film, and CDs/PVA composite film and (b) their comparison in the lower wavenumbers region.

To predict the chemical structure of the as-prepared sample, FTIR measurement was conducted and the result is shown in Fig. 2a. The broad peak observed at 3240 cm^−1^ can be attributed to O–H or N–H stretching vibrations. The presence of hydroxyl and amine groups not only makes the CDs hydrophilic but also increases their stability and dispersibility in water, which makes the CDs capable of medical applications such as bioimaging and photothermal therapy.^2,16,26^ The peak at 1621 cm^−1^ in the liquid CDs spectrum is associated with C=N and C=O stretching vibrations.^27,28^ The carboxylic functional group, C=O, is produced by the dehydration process of the –OH group and the –COOH group of citric acid.^29^ The absorption peak at 1058 cm^−1^ is characteristic of the C–O–C, epoxy group.^30^ Peaks at 1450 and 1360 cm^−1^ are ascribed to C=C and C–N groups, respectively, where the C=C is the main chemical functional group confirming that the sample is CDs.^28,31^ Based on our previous report, the presence of N–H, C=N, and C–N chemical groups indicates that CDs have nitrogen configurations such as pyrrolic-N, pyridinic-N, and graphitic-N.^24^ Meanwhile, the absorbance peak at ∼2900 cm^−1^ in the PVA and the CDs/PVA film spectra corresponds to the C–H bonds of the PVA structure. The spectrum of the CDs/PVA composite film is dominated by the PVA characteristics since the composition of CDs is only 4% v/v of PVA.

Fig. 2b shows the comparison of the FTIR spectra in the lower wavenumber region. It can be seen in the CDs/PVA spectrum that the intensity of the (CH)–CH_2_ bending peak of PVA is decreased and seen as a shoulder due to the rigid hydrogen bonding between CDs and PVA that limits the bending vibration of the (CH)–CH_2_ bonds. Meanwhile, the absorbance of C=N in the spectrum of CDs was observed at around 1578 cm^−1^, which confirms the presence of CDs in the composite. The absorbance of C=O bonds at around 1650 cm^−1^ and 1710 cm^−1^ are slightly broader due to the hydrogen bond formed by the interaction between CDs and PVA.^32,33^ These hydrogen bonds prevent direct collisions between the C=O groups and the O_2_ molecules present in the surrounding environment, thereby reducing the non-radiative recombination.^9^ The hydrogen crosslinking of CDs and PVA also increases the stability of the PVA structure.^6^ Besides, PVA also acts as a surface passivation agent for the CDs by forming a thin membrane around the surface of CDs to prevent the adhesion force between CDs and impurities from the environment.^18,34^

**Fig. 3:**
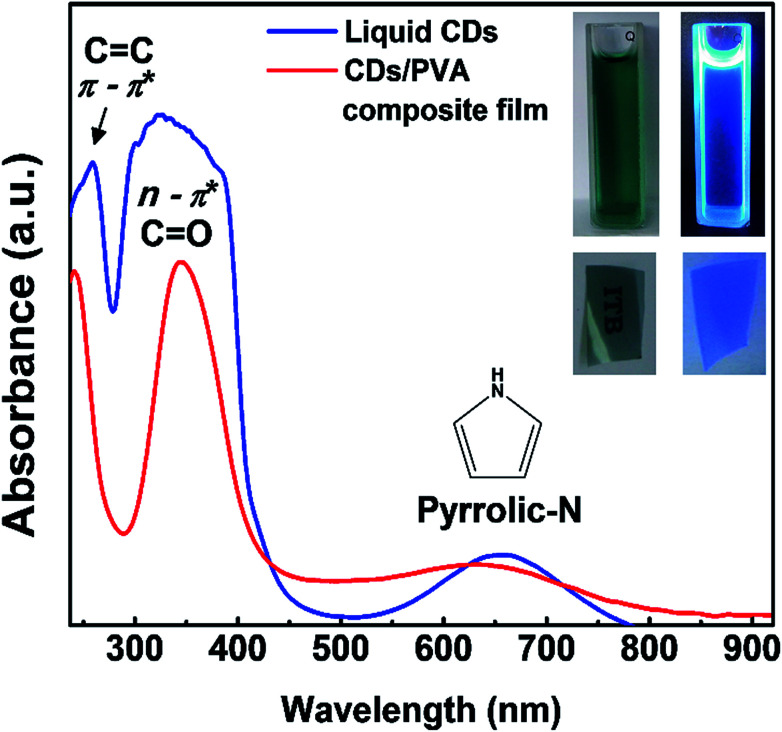
UV-vis absorption spectra of liquid CDs and CDs/PVA composite film; inset: photographs of liquid CDs and CDs/PVA composite film under daylight (up-left and down-left) and under 365 nm-UV light (up-right and down-right), respectively.

The color of liquid CDs under visible light was dark-blue and under UV light was bright blue as shown in Fig. 3. Meanwhile, the CDs/PVA composite film is transparent and emits in the blue region. Both as-prepared liquid CDs and CDs/PVA composite film could absorb UV and first NIR window simultaneously. The UV-vis spectra show that the liquid CDs and CDs/PVA composite film have three absorption peaks. The common UV absorption is characterized by two sharp peaks centered at 258 and 324 nm, which are ascribed to π → π* transition by the C=C aromatic group in the core of CDs and n → π* transition by the C=O group on the surface of CDs, respectively.^35^ Based on our previous report, the first NIR window absorption of liquid CDs centered at 650 nm might be generated by the pyrrolic nitrogen rich group on the surfaces.^23^ It was consistent with the FTIR results shown in Fig. 2 that confirm the abundance of C–N bonding on the CDs structure. The inherent abundance of delocalized electrons of the nitrogen rich pyrrolic group leads to high electron density. Then, the spreading of the delocalized electron wave enables the electrons to go through the forbidden transition. This is also supported by the first NIR window absorption exhibited by various particles with pyrrole structure on their surfaces.^36–38^ The optical properties of liquid CDs are quite sensitive to their environment.^5,23^ To enable the CDs for various applications, the liquid CDs were dispersed in PVA, which acted both as the matrix and the surface passivation agent. In the CDs/PVA films, no change in the UV absorption wavelength range could be observed. This was in good agreement with previous reports, thus confirming that PVA did not alter the UV absorption of CDs.^39,40^ However, there was a slight shift in the first NIR window absorption, which might have been induced by the interaction of PVA with the rich pyrrolic N on the CDs surfaces. To support this argument, we varied the concentration of CDs dispersed in PVA and their UV-vis spectra are shown in Fig. S1.† On varying the CDs concentration, there was no change in the absorption features except an increase in the absorption intensity with the increase in the CDs concentration. This indicates that varying the CDs concentration did not alter the interaction between CDs and PVA.

**Fig. 4:**
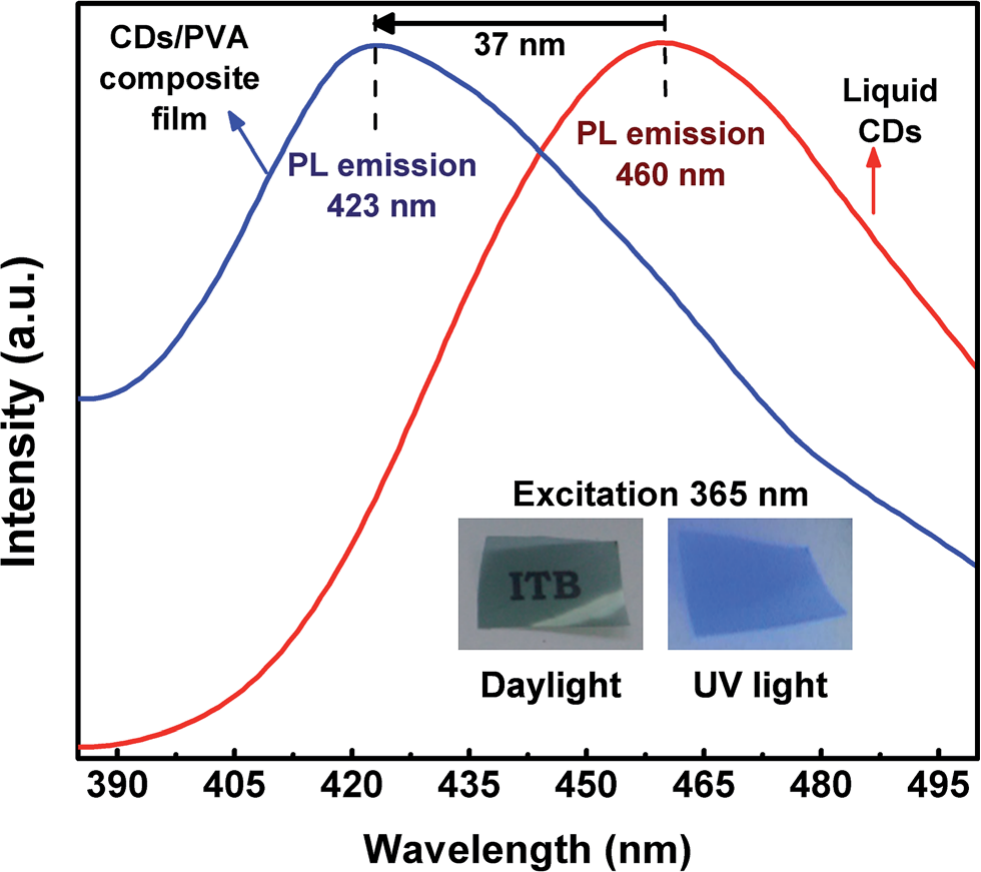
PL spectra of liquid CDs (red) and CDs/PVA composite film (blue).

The PL spectra of liquid CDs and CDs/PVA composite film are shown in Fig. 4. The graph shows that the PL spectrum of CDs/PVA composite film was shifted to the high-energy side (lower wavelength) known as blue-shift emission. The blue-shift emission of the CDs/PVA composite film was induced by the interaction between CDs and PVA. This interaction forms the hydrogen bonding that prevents direct collisions between C=O groups of CDs with the O_2_ molecules in the environment.^9^ PVA protects the CDs from non-radiative recombination that induced the red-shift emission of CDs causing the CDs quantum yield to decrease.^19,41^ Besides, PVA also successfully increases the quantum yield and lifetime of CDs.^19^ Under various excitation wavelengths, liquid CDs showed an excitation-dependent emission feature, while the emission of CDs/PVA composite film was excitation-independent as shown in Fig. S2 and S3.† Although both samples exhibited first NIR windows absorption, emission was not observed under the first NIR windows excitation. This indicates that the first NIR window did not generate the radiative transition into down conversion photoluminescence. However, the possibility of the up-conversion luminescence or photon–phonon conversion in the first NIR window is still open.

**Fig. 5:**
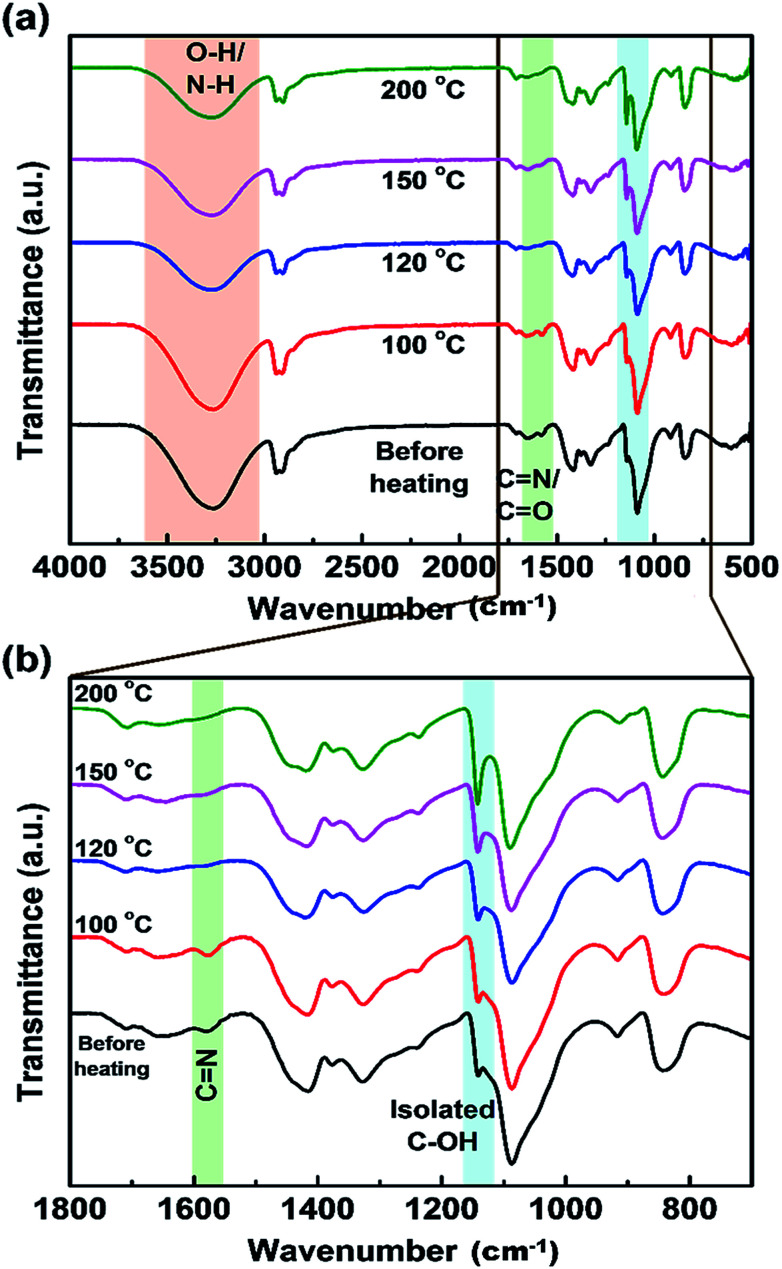
FTIR spectra of CDs/PVA films at different preheating temperatures in (a) a wide range of wavenumbers, and (b) the lower wavenumber region.

To further analyze the effect of PVA on the optical properties of CDs, the as-prepared CDs/PVA composite films were preheated at various temperatures (100, 120, 150, and 200 °C) for one hour. FTIR spectra in Fig. 5 shows that there is no difference between the spectra of the composite film before and after heating at 100 °C, suggesting that the composite was stable up to 100 °C. Decreased intensity of the peak of OH groups accompanied by peak shifting to higher wavenumbers is observed when the heating temperature increased from 100 to 200 °C (Fig. 5a). The peak intensity of the peak related to the C=N group in the CDs structure at around 1578 cm^−1^ also decreases after heat treatment at 120 °C and higher. Meanwhile, the spectrum in the lower wavenumber region (Fig. 5b) shows that the peak shoulder attributed to the (C–O)–C–OH groups at ∼1140 cm^−1^ become more prominent as that of the isolated C–OH bonds. These results indicate that the hydrogen bond between CDs and PVA is broken by thermal energy resulting in PL quenching of the CDs/PVA films.^20,33^ In addition, the PVA chains were also reported as a hindrance for CDs agglomeration caused due to intramolecular interaction, which is also a reason for PL quenching.^42^

It has been suggested that heat treatment can cause PL quenching of CDs, where one of the quenching mechanisms is the collision of C=O with O_2_ in the environment.^9^ This collision could be prevented by the hydrogen bonding, which is highly sensitive to the environmental temperature and pressure. Therefore, to confirm this speculation, the effect of hydrogen bonding as an inhibitor of PL quenching and absorption properties of the CDs/PVA films was evaluated by heating the CDs/PVA film at elevated temperatures in the range of 100 to 200 °C.

**Fig. 6:**
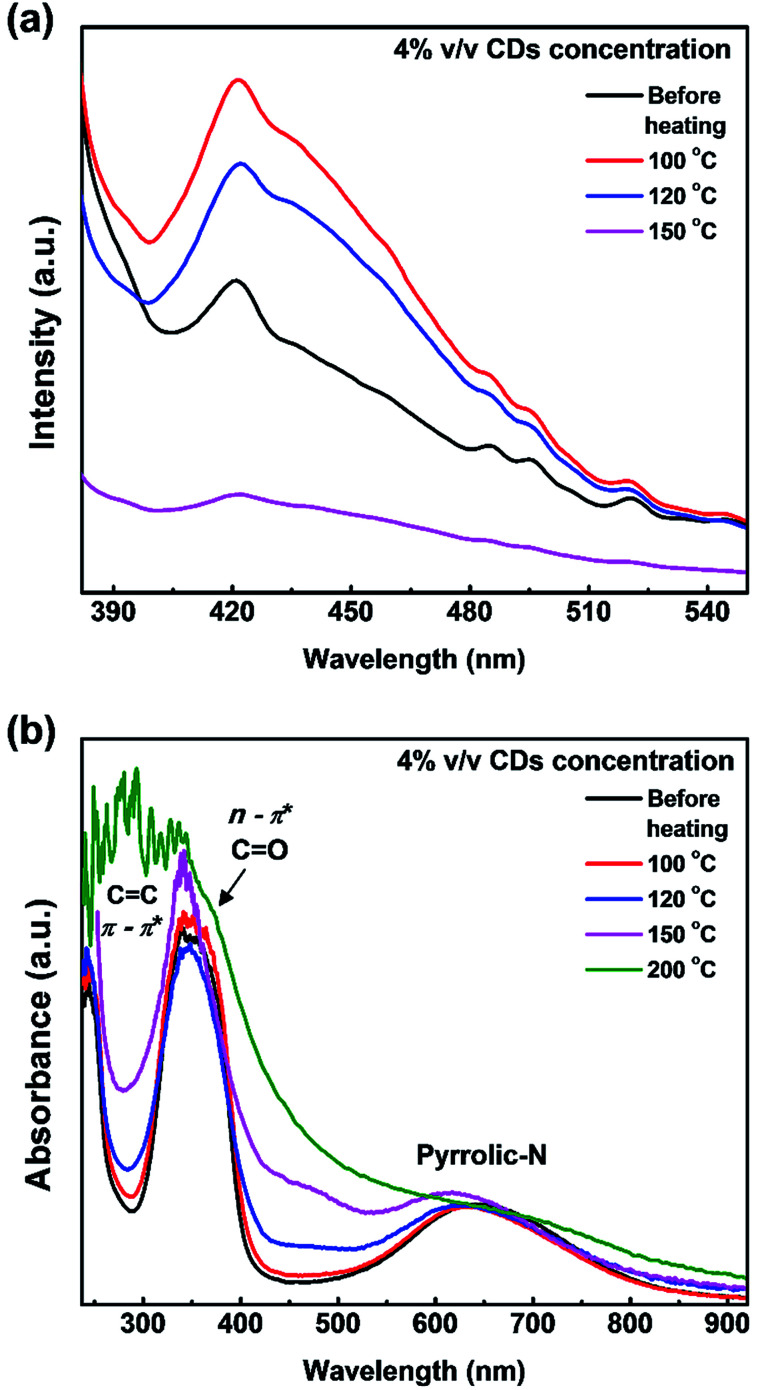
Effect of preheating treatment on the (a) PL intensity and (b) UV-vis spectra of CDs/PVA composite films.

The PL spectra (Fig. 6a) show that the luminescence intensity of CDs/PVA films decreases at heating temperatures of 120 and 150 °C. Another report proposed that temperature also has a quenching effect on CDs.^43^ These agree well with our previous report that the luminescence of CDs originates from the C–N configuration.^25^ The luminescence intensity of the films increased when the heating temperature was 100 °C (Fig. 6a). The increase in the luminescence intensity is supposedly due to the evaporation of water molecules from the environment of CDs, whereas H_2_O molecules can cause a quenching effect by enhancing the self-assembly of CDs to form aggregates.^5^ However, the luminescence intensity declined when heated at 120 °C or higher, which is most likely due to the removal of the hydrogen bond between CDs and PVA. These results are in a good agreement with the FTIR results, which showed that the hydrogen bond was maintained at 100 °C and started to break after 120 °C (Fig. 6).

Based on the UV-vis results in Fig. 6b, there are no significant changes in the UV and first NIR window absorption wavelengths for heat treatment up to 150 °C, which indicates that PVA was able to protect the CDs structure during the preheating process. A slight change in the first NIR window absorption wavelength, which corresponds to the pyrrolic-N structure is in good agreement with the FTIR results that show a decrease in the C=N bond transmission at high temperatures. Thus, it is confirmed that the origin of the first NIR window absorption is from the C–N groups on the surface of CDs. The absorption ability in the first NIR window is more important than the UV-vis absorption for applications in the medical field. Therefore, further study of this composite is important.

**Fig. 7:**
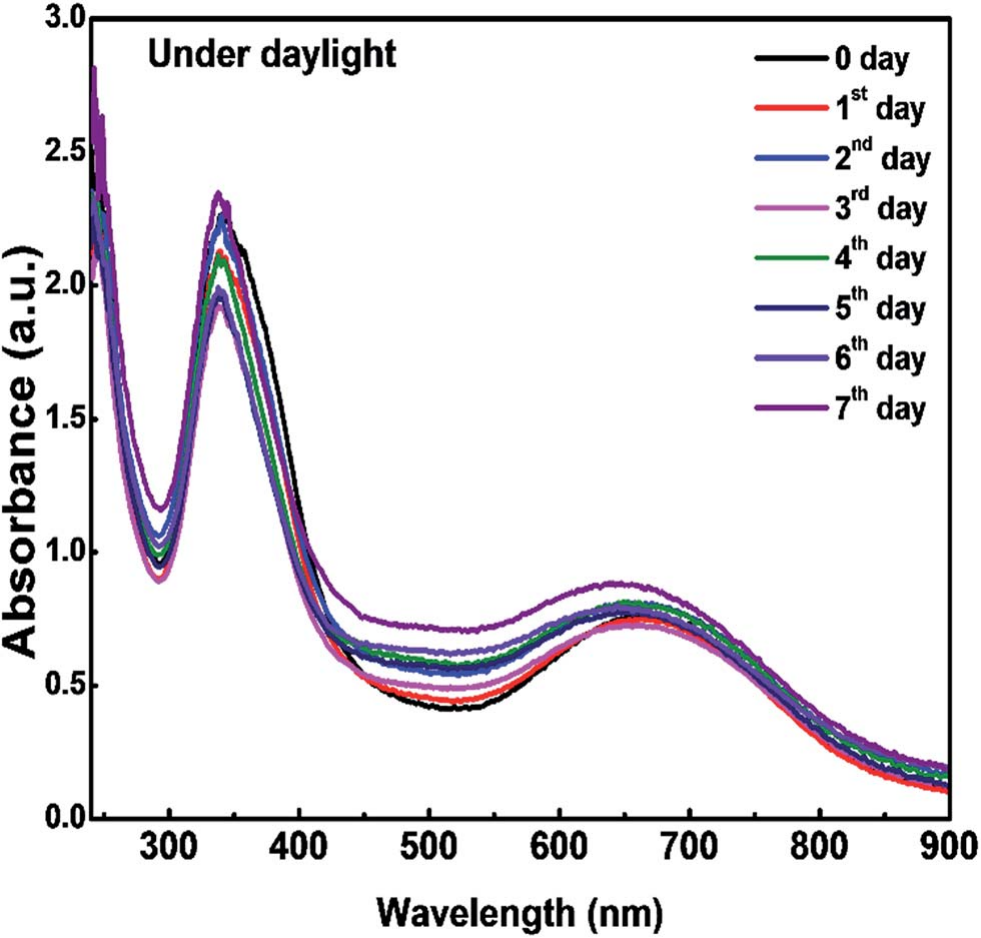
UV-vis spectra of CDs/PVA composite films at different aging times.

To study the stability of the CDs/PVA composite film, as-prepared films were stored under daylight for seven days and the absorbance intensity was measured every day. From the UV-vis spectra in Fig. 7, there is no apparent change in the absorption intensity. When compared with the liquid CDs in our previous report, the CDs/PVA composite films are more stable as the absorbance of the liquid CDs synthesized at 160 °C declined significantly within two days of aging time.^23^ This study shows that the hydrogen bonding between CDs and PVA successfully protects CDs from the direct collision between CD molecules and other molecules in the environment, and therefore, improves the stability of the CDs.

## Conclusions

CDs luminescent films that simultaneously absorb in the UV and first NIR window regions were successfully fabricated by the hydrothermal method coupled with PVA coating. FTIR spectra confirmed that nitrogen with pyrrolic-N, pyridinic-N, and graphitic-N configurations were incorporated into the CDs. PVA incorporation was indicated by the generation of C=N peak and the decrease in the intensity of (CH)–CH_2_ peak. UV-vis and PL spectra show that the CDs/PVA composite films can maintain the UV and first NIR window absorptions even after the preheating treatment up to 200 °C. Daylight treatment for seven days shows minimum changes in the UV-vis and PL spectra, which indicates that PVA successfully reduces the quenching effect of CDs.

## Conflicts of interest

There are no conflicts to declare.

## Supplementary Material

RA-009-C8RA09742A-s001
