# Supplementary material for: Simultaneous ultraviolet and first near-infrared window absorption of luminescent carbon dots/PVA composite film
Source: RSC Adv. 2019 Mar 6;9(13):7375–81. doi: 10.1039/c8ra09742a (PMC9061165; doi:10.1039/c8ra09742a)
Supplement: RA-009-C8RA09742A-s001 [file RA-009-C8RA09742A-s001.pdf]

Electronic Supplementary Material (ESI) for RSC Advances.
This journal is © The Royal Society of Chemistry 2019

Electronic Supplementary Information

Simultaneous ultraviolet and first near-infrared window absorption of luminescence carbon dots/PVA composite film

Melda Taspika,[a] Fitri Aulia Permatasari,[a] Bebeh Wahid Nuryadin,[b] Tirta Rona Mayangsari,[c,d] Akfiny Hasdi Aimon,[a] and Ferry Iskandar,[a,c*]

a. Department of Physics, Faculty of Mathematics and Natural Sciences, Institut Teknologi Bandung, Jalan Ganesha 10 Bandung 40132, Indonesia. E-mail: ferry@fi.itb.ac.id
b. Department of Physics, Universitas Negeri Islam Sunan Gunung Djati, Jalan A. H. Nasution Cibiru Bandung 40614, Indonesia.
c. Research Center for Nanosciences and Nanotechnology (RCNN), Institut Teknologi Bandung, Jalan Ganesha 10 Bandung 40132, Indonesia.
d. Department of Chemistry, Universitas Pertamina, Jl. Teuku Nyak Arief, Simprug, Jakarta 12220, Indonesia.

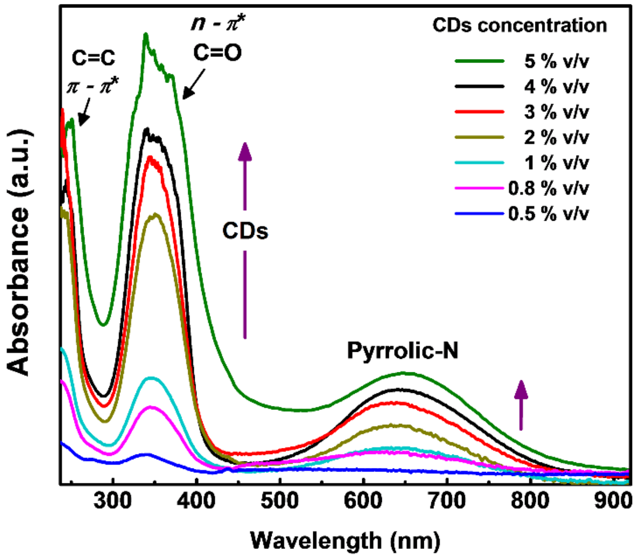

Fig. S1. UV-vis spectra of CDs/PVA composite films with different concentration of dispersed CDs in PVA.

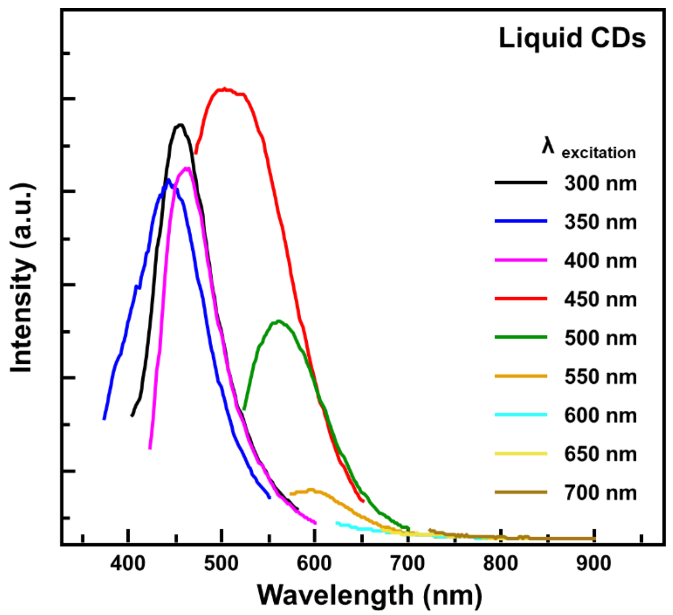

Fig. S2. Photoluminescence spectra of the liquid CDs under various excitation wavelength.

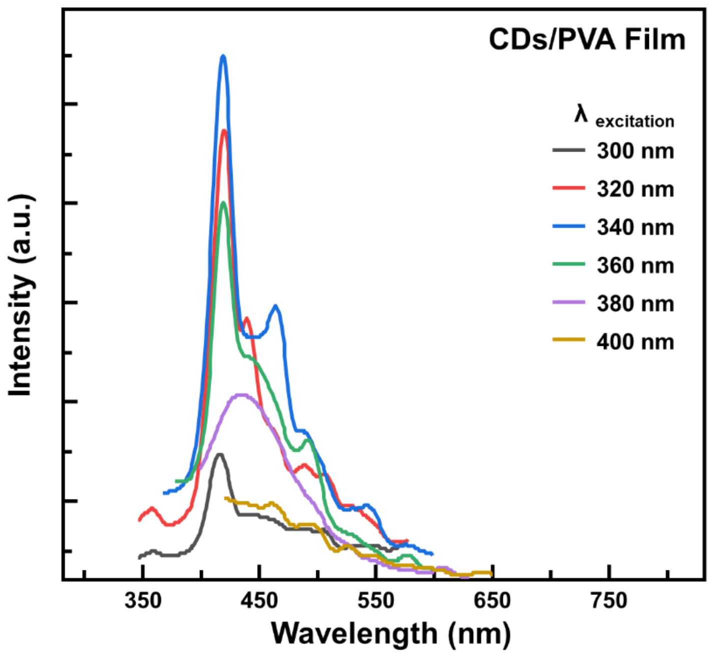

Fig. S3. Photoluminescence spectra of the CDs/PVA composite film with CDs concentration of 4% v/v under various excitation wavelength.
